# Patients with glycogen storage diseases undergoing anesthesia: a case series

**DOI:** 10.1186/s12871-017-0428-x

**Published:** 2017-10-06

**Authors:** Carmelina Gurrieri, Juraj Sprung, Toby N. Weingarten, Mary E. Warner

**Affiliations:** Department of Anesthesiology and Perioperative Medicine, Mayo Clinic, 200 First St SW, Rochester, MN 55905 USA

**Keywords:** General anesthesia, Glycogen storage disease, Perioperative complications

## Abstract

**Background:**

Glycogen storage diseases are rare genetic disorders of glycogen synthesis, degradation, or metabolism regulation. When these patients are subjected to anesthesia, perioperative complications can develop, including hypoglycemia, rhabdomyolysis, myoglobinuria, acute renal failure, and postoperative fatigue. The objective of this study was to describe the perioperative course of a cohort of patients with glycogen storage diseases.

**Methods:**

This is a retrospective review of patients with glycogen storage diseases undergoing anesthetic care at our institution from January 1, 1990, through June 30, 2015 to assess perioperative management and outcomes.

**Results:**

We identified 30 patients with a glycogen storage disease who underwent 41 procedures under anesthesia management. Intraoperative lactic acidosis developed during 4 major surgeries (3 liver transplants, 1 myectomy), and in all cases resolved within 24 postoperative hours. Lactated Ringer solution was used frequently. Preoperative and intraoperative hypoglycemia was noted in some patients with glycogen storage disease type I, all of which responded to administration of dextrose-containing solutions. No serious postoperative complications occurred.

**Conclusions:**

Patients with glycogen storage disease, despite substantial comorbid conditions, tolerates the anesthetic management without major complications. Several patients who experienced self-limited metabolic acidosis were undergoing major surgical procedures, during which acidosis could be anticipated. Close monitoring and management of blood glucose levels of patients with glycogen storage disease type I is prudent.

## Background

Glycogen storage diseases (GSDs) are disorders of the metabolism of glycogen which result from mutations in the genes involved in either the synthesis and degradation of glycogen or the regulation of glycogen metabolism. They have been categorized by number according to the recognition of the responsible enzyme defect [1]. The age at onset of disease varies from in utero to adulthood. Patients may have various clinical presentations, including exercise intolerance, myalgias, myoglobinuria, severe hypoglycemia, hepatomegaly, and cardiomyopathy. When subjected to anesthesia, these patients are at risk for the development of hypoglycemia, rhabdomyolysis, myoglobinuria, and acute renal failure [2–7]. The aim of this study was to characterize the perioperative course of patients with GSDs who underwent anesthetic management at a single tertiary medical center.

## Methods

This study was approved by the Mayo Clinic Institutional Review Board study, (Study ID 15–004738, approval date July 6, 2015, by Ellen Olson). Consistent with Minnesota Statute 144.335, we included only patients who had provided authorization for research use of their medical records.

We searched our institutional patient database to identify the records of patients with GSDs who underwent surgeries or procedures with anesthetic management at Mayo Clinic, Rochester, Minnesota, between January 1, 1990, and June 31, 2015. The diagnosis of GSD was made through clinical signs and symptoms and was confirmed by muscle biopsy, liver biopsy, or genetic testing. For all patients identified, the records were reviewed for demographics, preexisting comorbid conditions, surgical/procedural records, anesthetic type and course, and type of fluids used intraoperatively. We also reviewed preoperative, intraoperative, and postoperative laboratory values, with particular attention to arterial pH and serum glucose, and lactate levels as potential markers of metabolic decompensation. Hypoglycemia was defined as blood glucose concentrations of 70 mg/dL or lower [8]; and metabolic acidosis was defined as pH imbalance due to a reduction in bicarbonate (HCO3−) of 22 mmol/L or lower, which was not a result from secondary causes, e.g., chronic respiratory alkalosis. In addition, we reviewed postoperative course and patient disposition (eg, admission to general medical unit or intensive care unit, or outpatient procedure) and any complications within 30 postoperative days. Demographics and perioperative characteristics were summarized using descriptive statistics.

## Results

Our search of the database identified 30 patients with GSD who underwent 41 procedures under anesthesia management.

### Preoperative clinical characteristics

Table 1 shows the clinical characteristics of patients with GSD, which varied depending on the specific disorder. Patients with type I disorder (von Gierke disease) primarily had metabolic disorders consisting of hypoglycemia. Patients with type II (Pompe disease) and type V (McArdle disease) often had myopathy and muscle weakness. Patients with type II disorder often had respiratory failure related to myopathy or muscle weakness and heart failure from hypertrophic cardiomyopathy. Two of these patients (#13 and #15) underwent preoperative Holter monitoring: sinus tachycardia (fastest heart rate, 137 beats/min) was detected in patient #13, and ventricular tachycardia (fastest heart rate, 163 beats/min) was detected in patient #15. Cardiac manifestations were also present in the patient with type 0 disorder. Patients with other types of GSD typically had muscle weakness, myopathy, or hepatosplenomegaly, or a combination of these.

**Table 1:** Clinical characteristics of study patients with glycogen storage disease

	Clinical presentation	
Pt	Neurologic/Muscular	Other
GSD type 0		
1	Muscle weakness	OSA; hypertrophic cardiomyopathy; hypertension
GSD type I (von Gierke)		
2	Muscle weakness; gait abnormalities	Lactic acidosis; hypoglycemia; hepatic adenoma
3	None reported	Hypoglycemia
4	None reported	Hypertension; hypoglycemia; lactic acidosis
5	Seizures	Hyperuricemia; lactic acidosis; hypoglycemia
6	Developmental delay; velopharyngeal insufficiency	Hypoglycemia
7	Fatigue, muscle weakness	Hypoglycemia
GSD type II (Pompe)		
8	Severe scoliosis; muscle weakness; wheelchair-bound	Respiratory failure (BiPAP); restricted lung disease
9	Muscle weakness; hypotonia	None reported
10	Muscle weakness	Respiratory failure (BiPAP); pulmonary hypertension; CHF; dilated cardiomyopathy; atrial fibrillation
11	Muscle weakness	None reported
12	Muscle weakness	CHF; dilated cardiomyopathy
13	Muscle weakness	Respiratory failure (BiPAP)
14	Muscle weakness	Dyspnea; hypertension
15	None reported	Hypertrophic cardiomyopathy; ventricular tachycardia
GSD type IV (Andersen)		
16	Developmental delay	Hepatosplenomegaly
GSD type V (McArdle)		
17	Muscle weakness; cervical radiculopathy	None reported
18	Muscle stiffness; increased serum creatine kinase	None reported
19	Muscle weakness	None reported
20	Muscle weakness	None reported
21	Muscle cramps; exercise intolerance	None reported
22	Exercise intolerance	None reported
23	Muscle weakness	None reported
24	Muscle weakness and stiffness	None reported
25	Muscle weakness	None reported
26	Episodes of rhabdomyolysis; exercise intolerance	None reported
27	Muscle weakness	Hypertension
GSD type VI (Hers)		
28	None reported	Lactic acidosis
GSD type VII (Tarui)		
29	Muscle weakness	None reported
Unidentified, likely glycolytic defect		
30	Proximal muscle myopathy	Hypoglycemia

*Abbreviations*: *BiPAP* bilevel positive airway pressure, *CHF* congestive heart failure, *GI* gastrointestinal, *MH* malignant hyperthermia, *OSA* obstructive sleep apnea, *Pt* patient

### Anesthesia course and Intraoperative fluid administration

Case mix and anesthetic management is summarized in Table 2. Twenty-two procedures were outpatient based. Twenty-eight procedures were performed under general anesthesia, of which 23 were performed with endotracheal intubation and 5 with a laryngeal mask airway. The other 13 procedures were performed under monitored anesthesia care. Most patients underwent either diagnostic biopsies (*n* = 20) or minor procedures (*n* = 13) (e.g., dilation and curettage, reduction mammoplasty, gastric tube placement). The other patients (*n* = 8) underwent extensive operations: liver transplant (*n* = 3), myectomy (n = 1), Whipple procedure (n = 1), and spinal fusion (n = 3).

**Table 2:** Case mix, surgical and anesthetic characteristics in patients with glycogen storage disease

Pt	Age^a^ at Surgery, y	Surgery/Anesthesia duration, min/Airway/Anesthetic Agents	Fluid used	Disposition
GSD type 0				
1	51–60	Muscle biopsy/120,P	LR	Outpatient
GSD type I (Von Gierke)				
2	19–30	Liver transplant/360/P, C, S, Iso	NS; D5W; PRBC; Cryo; Albumin	ICU
3	11–18	Liver transplant/300/P, C, Iso	NS; D5W; Albumin	ICU
4	6–10	Liver transplant/330/P, V, Iso	NS; D5NS; PRBC; Albumin	ICU
5	1–2	Muscle biopsy/120/Sev, N_2_O	D5NS	Outpatient
	1–2	Gastric tube placement/60/P, At, Iso	D10NS	Ward
6	<1	Gastric tube placement/100/P, At, Sev	LR; D10NS	Ward
	3–5	Tonsillectomy/110/P, V	LR; D10NS	Ward
	3–5	Myringotomy/ 100/P	LR; D10NS	Ward
7	11–18	Rectal abscess I&D/70/At, STP, N_2_O	D10W	Outpatient
	11–18	Abscess I&D/120/P, At, N_2_O	LR; D10W	Outpatient
GSD type II (Pompe)				
8	11–18	Anterior spinal fusion/300/STP, Iso, Sev, P	LR; PRBC; FFP; Platelets; Hetastarch; Albumin	ICU
	11–18	Posterior spinal fusion/730/P, Sev, N_2_O	LR	ICU
9	<1	Muscle biopsy/75/P, N_2_O	LR	Outpatient
10	41–50	Muscle biopsy/MAC/60, P	LR; NS	Ward
	41–50	Tracheostomy/80/E, S, Sev	LR	ICU
11	51–60	Muscle biopsy/MAC/90 mask, P	NS	Outpatient
12	31–40	Myocardial biopsy/MAC/90, Mz, AL	LR	Outpatient
	31–40	Parathyroidectomy/120/E, V, Iso	LR	Ward
13	41–50	Muscle biopsy/MAC/60,P	LR	ICU
14	41–50	Muscle biopsy/MAC/120/ Mask, P	NS; LR; Albumin	Outpatient
15	3–5	Myectomy (ICD)/ CPB/280,Sev, V, Iso	LR	ICU
	3–5	Sternal wire removal/180/E, RO, Sev		Ward
Glycogen storage disease type IV (Andersen)				
16	1–2	Liver biopsy/MAC/60/ Mask, Sev	LR	Outpatient
	1–2	Myringotomy/GA/60/Sev, N_2_O	None	Outpatient
17	61–70	Cervical discectomy/200, P, RO		Ward
Glycogen storage disease type V (McArdle)				
18	11–18	Muscle biopsy/60)/ LMA/Sev, N_2_O	LR	Outpatient
19	6–10	Muscle biopsy/60/P, Sev, N_2_O	LR	Outpatient
20	41–50	Whipple /GA /360/P, S, V, Iso	LR	Ward
21	41–50	Muscle biopsy/MAC/60/ Mask/P	LR	Outpatient
22	11–18	Muscle biopsy/MAC/90/ Mask/P, Sev	LR	Outpatient
23	19–30	Muscle biopsy/ MAC/105/ Mask, P	LR	Outpatient
24	19–30	Muscle biopsy/ MAC/60/ Mask, P	LR	Outpatient
25	6–10	Muscle biopsy/70/ LMA, P	LR	Outpatient
26	19–30	Muscle biopsy/ MAC/120/ Mask, P	LR	Outpatient
27	>70	Muscle biopsy/MAC/60/ Mask, P	LR	Outpatient
GSD type VI (Hers)				
28	1–2	Liver biopsy/105/Sev, RO	D5NS	Outpatient
GSD type VII (Tarui)				
29	61–70	Muscle biopsy/MAC/210/Mask, P	LR	Outpatient
Unidentified, likely glycolytic defect				
30	31–40	Dilation & curettage/60/LMA/P, N_2_O	LR	Outpatient
	31–40	Mammoplasty/240/P, V, N_2_O	LR	Ward
	31–40	Breast hematoma evacuation/90/LMA/P, N_2_O	LR	Ward

*Abbreviations*: *At* atracurium, *BiPAP* bilevel positive airway pressure, *C* cisatracurium, *CPB* cardiopulmonary bypass, *D10NS* 10% dextrose with 0.9% normal saline, *D10W* 10% dextrose with water, *D5NS* 5% dextrose with 0.9% normal saline, *D5W* 5% dextrose with water, *E* etomidate, *F* female, *GA* general anesthesia, *ICD* implantable cardioverter-defibrillator; *ICU* intensive care unit, *I&D* incision and drainage, *Iso* isoflurane, *LMA* laryngeal mask airway, *M* male, *Mz* midazolam, *MAC* monitored anesthesia care, *N*_*2*_ *O* nitrous oxide, *NA* not available, *NS* 0.9% normal saline, *P* propofol, *PRBC* packed red blood cells transfusion, *Pt* patient, *RO* rocuronium, *S* succinylcholine, *Sev* sevoflurane, *STP* sodium thiopental, *V* vecuronium

^a^Age is reported as a range (in year): <1 year; 1–2; 3–5; 6–10; 11–18; 19–30; 31–40; 41–50; 51–60; 61–70; >70

Nondepolarizing neuromuscular blocking drugs were administered in 16 patients, and succinylcholine was used in 3, without adverse effects, including patients with Pompe disease and preexisting muscle weakness. Propofol infusions were used in 14 cases. Lactated Ringer’s solution was used in 33 procedures (80%), but patients with a history of hypoglycemia were intraoperatively administered dextrose-containing solutions, including all patients with type I. Among patients with type I, 2 of them (#2 and #5) had hypoglycemia preoperatively and 3 (#3, #4, and #15) had borderline low glucose levels (71–75 mg/dL). Patients #2 and #3 developed hypoglycemia during orthotopic liver transplant surgery (59 and 34 mg/dL, respectively). Perioperative mild metabolic acidosis developed in 4 patients undergoing orthotopic liver transplant and myectomy, which resolved within 24 postoperative hours.

### Postoperative course

No clinically significant postoperative complications occurred among the group (Table 2). There were no unexpected intensive care unit admissions, and all patients admitted to the intensive care unit were discharged to a regular postoperative ward within 24 h.

## Discussion

The most important observation of this study was that patients with GSDs, despite having substantial comorbid conditions, tolerated surgery and anesthesia without major complications. Self-limited, transient, metabolic acidosis developed in several patients undergoing major surgical procedures, during which it may be expected however, it remains unclear whether underlying metabolic disorder was a contributing factor. Another important observation was that hypoglycemia was present in some patients with GSD type I. In this subset of patients, it is prudent to use glucose-containing fluids and closely monitor glucose levels.

### GSD type I (von Gierke disease)

Patients with type I are at increased risk for perioperative metabolic and homeostatic derangements, particularly hypoglycemia. These abnormalities are mainly related to a defect of either glucose-6-phosphatase or glucose-6-phosphatase transporter, which impairs the production of glucose via either gluconeogenesis or glycogenosis. Accordingly, frequent monitoring of blood glucose is recommended during the perioperative period [1, 9–11] especially during major procedures. In our series, patients with hypoglycemia were administered dextrose-containing solutions during their procedures. Patient #4 had intraoperative hypoglycemia (34 mg/dL), he received a dextrose solution infusion, and all subsequent glucose checks were above 100 mg/dL. This patient underwent liver transplant, which in itself can contribute the glucose metabolism impairment and can potentially cause hypoglycemia. Patients with type I disease are also prone to the development of organic acidemias; in this series, the 3 patients who underwent liver transplant were known to have lactic acidosis (patients #2, #3, and #4). Lactate ringer solution (LR) was used for 7 minor procedures without incident, albeit in patients without known organic acidemias, and in conjunction with dextrose-containing solutions.

Patients with type I are also at risk for the development of pancreatitis secondary to hypertriglyceridemia [1, 12]. Bustamante and Appachi [11] reported a case of pancreatitis in a 4-year-old girl with type I undergoing general anesthesia for tonsillectomy with propofol infusion. In our series, the 3 patients undergoing liver transplant received propofol infusion as a part of their anesthetic, with an average of 12 h’ duration, withoutadverse effects. Patient with GDS type I may also have coagulation abnormalities arising from acquired von Willebrand factor abnormalities, and the perioperative use of desmopressin advocated to facilitate hemostasis [13, 14]. However, in our cohort the perioperative records did not indicate coagulopathy.

### GSD type II (Pompe disease)

Patients with type II may have marked hypertrophic cardiomyopathy and an increased risk of dysrhythmia [15, 16]. Cases of intraoperative cardiac arrest have been described [17, 18]. Therefore, preoperative electrocardiography and echocardiography should be considered to assess both cardiac rhythm and function. Wang [15] described ventricular and supraventricular dysrhythmias in 9 infants with type II disease soon after the induction of general anesthesia with propofol and/or sevoflurane. Two of these patients died intraoperatively as a result of dysrhythmias. In our series, preoperative electrocardiography was performed in most patients with Pompe disease, and 2 of our patients (#13 and #15) undergoing muscle biopsy and septal myectomy had preoperative echocardiography and Holter monitoring. No perioperative dysrhythmias were noted in this subset of patients.

Type II disease is also known for skeletal muscle myopathies. These may lead to weakness of the respiratory muscles, which further leads to chronic respiratory failure [19] and increased risk of postoperative respiratory complications [20]. Indeed, 4 of our patients (#8, #10, #13, and #14) had known respiratory symptoms preoperatively. One patient (#10) underwent elective tracheostomy for management of chronic hypercarbic respiratory failure. Fortunately, none of our patients had acute worsening of respiratory symptoms postoperatively. Muscle relaxation with nondepolarizing agents should be used cautiously because residual weakness could be poorly tolerated. However, these drugs were used in 3 cases (#10, #12, and #15) without incident.

### GSD type V (McArdle disease)

Patients with type V disease may have rhabdomyolysis, myoglobinuria, and acute renal failure [2]. Cases of acute renal failure have been reported after strenuous or vigorous exercise [3, 4] and after an asthmatic attack [5]. Cases of massive rhabdomyolysis and myoglobinuria also have been observed after a diagnostic forearm ischemic work test using a tourniquet [2, 21]. Indeed, particular attention should be paid to intraoperative positioning because improper positioning can increase the risk of rhabdomyolysis. In our series, most patients with type V had a chronically high creatine kinase level, but patients had no abnormal temperature fluctuations, signs of hypermetabolism, or signs of rhabdomyolysis in the perioperative period. In this study, potential malignant hyperthermia triggers—volatile anesthetics and succinylcholine—were used in 4 cases (#18, #19, #20, and #22) with no clinical complications observed. Despite reported suspected cases of malignant hyperthermia in the type V disease [2, 22, 23], the U.S. Malignant Hyperthermia Association has stated that there is no association between any type of GSD and malignant hyperthermia [24].

### GSD type 0, IV, VI, and VII

To our knowledge, there are no reported cases of patients with type 0, IV, VI, or VII undergoing anesthesia who had perioperative complications related to their disease. It is known that types 0, IV, and VI affect the liver and that patients might experience mild hypoglycemia, whereas type VII primarily affects the muscle, which results in muscle weakness and myalgia. Any potential challenges related to anesthesia care in these patients are still uncertain. In our case series, the 4 patients with type IV, VI, VII, and 0 (#16, #28, #29 and #30, respectively), who underwent liver biopsy in the first 2 cases and muscle biopsy in the other 2, tolerated anesthesia well with no major perioperative complications. They were discharged the day of surgery. Because of the lack of previous reports and the limitations of our case series, no definitive recommendations can be made regarding anesthesia with these 4 GSD types. Further larger studies are warranted.

### Limitations

This study has all the inherent limitations of a retrospective study design. Moreover in our cohort none of the patients included in the study underwent regional anesthesia, thus our experience with anesthetic technique is limited.

## Conclusions

In our study, patients with various types of GSD undergoing anesthesia did not experience complications that could be attributed to anesthesia, per se. Hypoglycemia must be prevented in patients with type I (von Gierke disease), and thorough assessment of cardiac and pulmonary function is needed in type II (Pompe disease). Patients with type I (von Gierke disease) are also prone to the development of organic acidemias, but we did not encounter severe acid-base disturbances despite intraoperative administration of LR in some patients.

## Abbreviations

GSD: Glycogen storage disease
LR: Lactated Ringer solution
